# Solvothermal Synthesis of Hierarchical Colloidal Nanocrystal Assemblies of ZnFe_2_O_4_ and Their Application in Water Treatment

**DOI:** 10.3390/ma9100806

**Published:** 2016-09-29

**Authors:** Peizhi Guo, Meng Lv, Guangting Han, Changna Wen, Qianbin Wang, Hongliang Li, Xiu Song Zhao

**Affiliations:** Institute of Materials for Energy and Environment, State Key Laboratory Breeding Based of New Fiber Materials and Modern Textile, School of Materials Science and Engineering, Qingdao University, Qingdao 266071, China; lm199133@126.com (M.L.); kychgt@163.com (G.H.); qsdwcn256@163.com (C.W.); material_wqb@163.com (Q.W.); lhl@qdu.edu.cn (H.L.); chezxs@qdu.edu.cn (X.Z.)

**Keywords:** nanocrystal assembly, solvothermal synthesis, magnetism

## Abstract

Hierarchical colloidal nanocrystal assemblies (CNAs) of ZnFe_2_O_4_ have been synthesized controllably by a solvothermal method. Hollow ZnFe_2_O_4_ spheres can be formed with the volume ratios of ethylene glycol to ethanol of 1:4 in the starting systems, while solid ZnFe_2_O_4_ CNAs are obtained by adjusting the volume proportion of ethylene glycol to ethanol from 1:2 to 2:1. Magnetometric measurement data showed that the ZnFe_2_O_4_ CNAs obtained with the volume ratios of 1:2 and 1:1 exhibited weak ferromagnetic behavior with high saturation magnetization values of 60.4 and 60.3 emu·g^−1^, respectively. However, hollow spheres showed a saturation magnetization value of 52.0 emu·g^−1^, but the highest coercivity among all the samples. It was found that hollow spheres displayed the best ability to adsorb Congo red dye among all the CNAs. The formation mechanisms of ZnFe_2_O_4_ CNAs, as well as the relationship between their structure, crystallite size, and properties were discussed based on the experimental results.

## 1. Introduction

Spinel ferrites have been widely studied in recent years in many important fields, such as clinical and biomedical fields [1], microwave adsorption [2], optoelectronic devices [3], catalysis [4], and drug loading materials [5] due to their excellent physical and chemical properties. Diverse methods have been developed for the synthesis of ferrite materials, such as sol–gel techniques [6], hydrothermal/solvothermal method [7], and co-precipitation [8], etc. Among them, the solvothermal method has attracted more and more attention to the synthesis of targeted ferrite with high yield, narrow size distribution, and controlled morphology [9,10]. For example, bismuth ferrite microcrystals with controlled morphologies were obtained hydrothermally and can be used as the photocatalyst for the degradation of Congo red under visible light irradiation [11]. Nickel ferrite nanoparticles with the sizes from 6 to 170 nm were synthesized by simply adjusting the experimental parameters [12].

Among the spinel ferrites, zinc ferrite has brighter prospects due to their particular properties in many application fields, such as anode materials for lithium-ion batteries [13], gas sensors [14], magnetic materials [15], and catalytic materials [16,17]. For example, zinc ferrite with high stability and magnetic recoverability was synthesized using sol polyvinyl alcohol as surfactant, and was studied as a catalyst for the reduction of nitroarences [18]. ZnFe_2_O_4_ nanoparticles, which displayed enhanced gas sensing performance to acetone, were successfully synthesized by hydrothermal technology in the absence of structure directing agents or surfactant [19]. Hierarchical shuttle-shaped mesoporous ZnFe_2_O_4_ microrods with enhanced lithium storage for advanced Li-ion batteries were obtained using a two-step synthetic strategy [20]. However, it is still a great challenge to find a facile way to controllably synthesize hollow and even hierarchical structures of zinc ferrite nanomaterials.

In this paper, spherical colloidal nanocrystal assemblies (CNAs) of ZnFe_2_O_4_ with the mean diameter in the range of 100–300 nm have been synthesized via a solvothermal method, which were formed by the in situ self-assembly of small ZnFe_2_O_4_ nanoparticles. Among the CNAs, hollow ZnFe_2_O_4_ spheres can be prepared when the volume ratio of ethylene glycol to ethanol (EG/Et) was no more than 1:4, while solid CNAs were obtained when the EG/Et volume ratios were no less than 1:2. It was found that ZnFe_2_O_4_ CNAs showed unique magnetic properties, and could be used as effective absorbents of Congo red.

## 2. Results

### 2.1. Morphology and Structure

**Figure 1:**
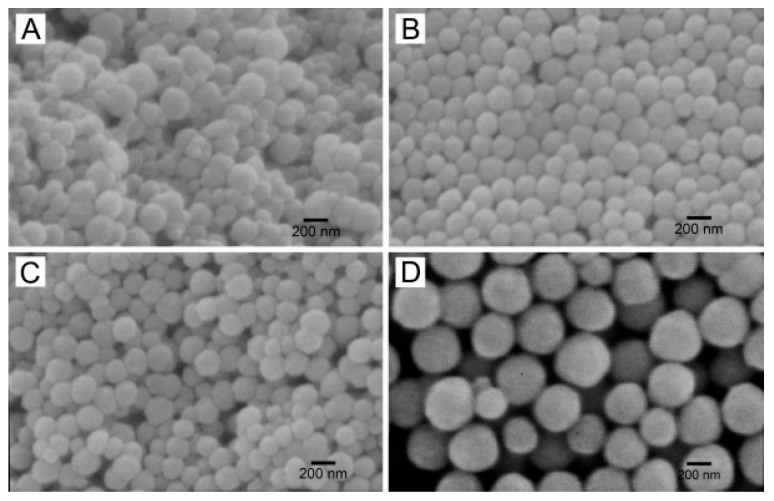
Scanning electron microscope (SEM) images of ZnFe_2_O_4_ colloidal nanocrystal assemblies (CNAs): (**A**) CNA1 (EG/Et = 1:4); (**B**) CNA2 (EG/Et = 1:2); (**C**) CNA3 (EG/Et = 1:1); and (**D**) CNA4 (EG/Et = 2:1).

**Figure 2:**
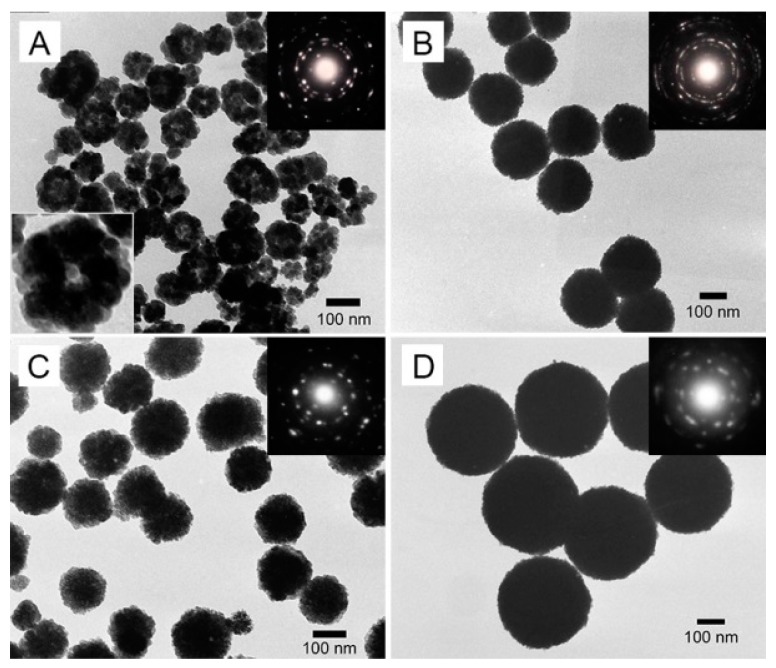
Transmission electron microscopy (TEM) images of ZnFe_2_O_4_ CNAs: (**A**) CNA1; (**B**) CNA2; (**C**) CNA3; and (**D**) CNA4.

The morphology and microstructure of the as-made ZnFe_2_O_4_ CNAs were investigated by scanning electron microscope (SEM) and transmission electron microscopy (TEM) measurements, as shown in Figure 1 and Figure 2, respectively. ZnFe_2_O_4_ CNAs obtained from the synthesis systems are abbreviated as CNA1, CNA2, CNA3, and CNA4, respectively, with the different EG/Et volume ratios of 1:4, 1:2, 1:1 and 2:1. As depicted in Figure 1, all of the samples showed a spherical-like shape, and their sizes increased from about 100 to 300 nm with the increment of ethanol content in the synthetic systems. Among them, CNA1 spheres with an average size of 120 ± 30 nm showed a relatively more rough edge compared to the other three samples (Figure 1A). It can be seen from Figure 1B,C that CNA2 and CNA3 had a narrow size distribution, with the diameter sizes of 130 ± 30 and 150 ± 25 nm, respectively, with a significantly increased value of 300 ± 50 nm for CNA4 (Figure 1D). Therefore, the size and morphology of these CNAs spheres were affected by the composition of the solvent. Namely, the sizes of ZnFe_2_O_4_ CNAs were greatly increased by gradually adding the proportion of ethylene glycol to ethanol in the synthetic systems.

Typical TEM images of ZnFe_2_O_4_ CNAs are shown in Figure 2. As depicted in Figure 2A, CNA1 exhibited a hollow structure with a relatively broad size distribution, the size of which was similar to that observed from the SEM image (Figure 1A). An enlarged image of a single CNA1 sphere is shown in the left-bottom of Figure 2A, which clearly denoted the formation of a hollow structure. However, the other three assemblies possessed well-separated submicrometer solid spherical structures. The corresponding sizes of CNA2, CNA3, and CNA4 were about 130, 150, and 300 nm, respectively, which were in good accord with those observed from SEM images (Figure 1B–D). The crystalline nature of the CNA samples can be generally deduced by the selected area electron diffraction (SAED) patterns inserted in the top right corner of Figure 2. Taking the SEAD pattern of CNA1 as an example (as presented in the inset of Figure 2A), it was not a typical single crystal lattice according to the diffraction spots as recorded on an isolated circle and the succedent featured arcs, which indicated that CNA1 was formed by the assembly of small primary nanoparticles with slight misalignments [21]. The alignment of adjacent primary particles can be understood as the consequence of the oriented attachment and subsequent aggregation of original crystallites and crystal growth during the synthetic process [22]. Similar results could be derived from other samples based on the SAED patterns, although the CNA spheres have different sizes. It should be noted that the diffraction spots in the SAED patterns of CNA2 and CNA4 are obviously wider than those of CNA1 and CNA3, indicating that the self-assembly of ZnFe_2_O_4_ nanoparticles in CNA2 and CNA4 were obviously more ordered or compacted compared with the other two samples. Further evidence for the morphologies of CNAs may be obtained from Figure 2. Namely, the surfaces of CNA2 and CNA4 were much smoother than those of CNA1 and CNA3. These were essential to the following results of X-ray diffraction (XRD), magnetic, and physicochemical measurements.

### 2.2. X-ray Diffraction (XRD) Analysis

**Figure 3:**
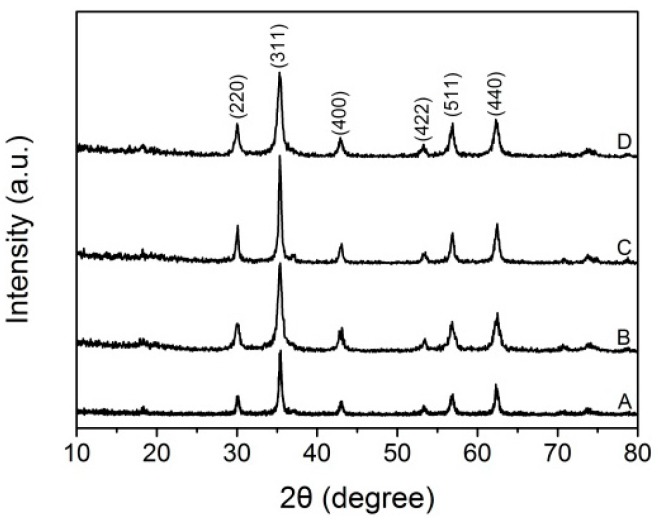
X-ray diffraction (XRD) patterns of ZnFe_2_O_4_ CNAs: (**A**) CNA1; (**B**) CNA2; (**C**) CNA3 and (**D**) CNA4.

Figure 3 shows the XRD patterns of the four CNA samples obtained at a temperature of 200 °C. Distinct diffraction peaks can be observed for all these samples, which matched well with the cubic spinel structure of ZnFe_2_O_4_ (JCPDS No. 77-0011). The compositions of Zn/Fe ratios were also confirmed by inductively coupled plasma-optical emission spectrometry (ICP-OES) measurement, and the results were all close to 1/2, verifying the stoichiometric nature of ZnFe_2_O_4_. The peaks observed at 2θ degrees of 30.2, 35.5, 42.9, 53.3, 56.8, and 62.3 in the XRD patterns respectively corresponded to the (220), (311), (400), (422), (511), and (440) planes of spinel ZnFe_2_O_4_. It can also be found that the widths of the diffraction peaks were relatively large, indicating that the crystallite sizes of ZnFe_2_O_4_ nanoparticles in all the CNAs were small. Furthermore, the diffraction peaks of CNA3 were sharper and narrower than any one of others, which meant that CNA3 had the largest crystallite size among all the samples. According to the Scherrer equation, the crystallite size of these ZnFe_2_O_4_ samples—taking the (311) peak as an example—can be calculated to be 20.4, 15.8, 25.3, and 15.5 nm for CNA1, CNA2, CNA3, and CNA4, respectively. These results further confirmed that all of the submicrometer spheres were formed from the assembly of primary ZnFe_2_O_4_ nanocrystals.

### 2.3. Evolution of the Intermediates

**Figure 4:**
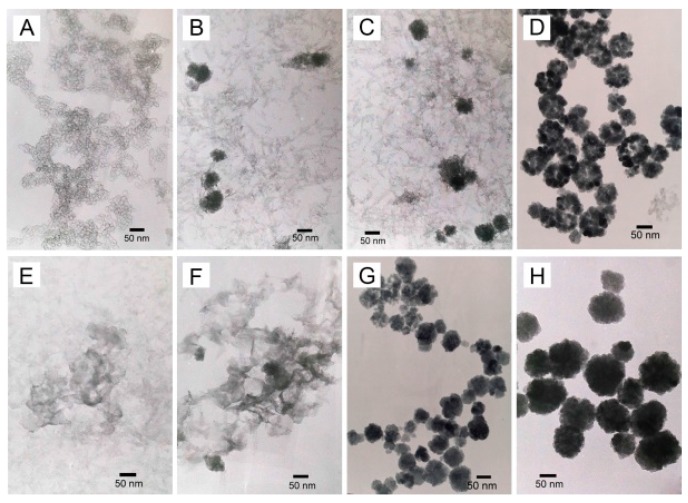
TEM images of the intermediates obtained from the synthesis systems for CNA1 (**A**–**D**) and CNA3 (**E**–**H**) at the synthetic times of 0.5 h (**A**,**E**), 1 h (**B**,**F**), 2 h (**C**,**G**) and 4 h (**D**,**H**).

In order to study the formation process of ZnFe_2_O_4_ hollow spheres and solid spheres, the intermediate products synthesized at different reaction times were collected and characterized by XRD (Figure S1) and TEM (Figure 4) measurements. CNA3 was selected as a representative of the ZnFe_2_O_4_ solid CNAs systems. Figure 4A presents the TEM image of the product obtained at 0.5 h from the hollow sphere synthetic system, and a bubble-like structure can be observed. Meanwhile, the ZnFe_2_O_4_ phase was already obtained based on the corresponding XRD pattern (Figure S1A). When the synthesis time extended to 2 and 4 h, it can be seen that thin wire nanostructures and aggregate particles with sizes of about 50 nm were formed, as shown in Figure 4B,C, respectively. The obvious peaks in the XRD patterns indicated that zinc ferrite was formed at this time (b and c in Figure S1A). Clearly, ZnFe_2_O_4_ nanoparticle aggregates were observed with sizes of 30–100 nm. As depicted in Figure 4D, submicrometer spheres and some hollow spheres were formed, while thin wire structures disappeared. The corresponding sharp peaks observed in the XRD pattern can be indexed to cubic ZnFe_2_O_4_ phase (JCPDS No. 77-0011). It was speculated that due to the self-assembly and secondary growth of ZnFe_2_O_4_ nuclei, hollow structures were finally obtained with the solvothermal time up to 12 h (Figure 2A), which was consistent with the Ostwald ripening process [23,24]. The spherical aggregates were first developed with the aggregation of original crystallites due to the minimization of interfacial energy, and then the crystallites migrated outward gradually because of the energy difference between them during a recrystallization process. In the presence of large amounts of ethanol, the hollow spheres were finally formed when the core region successively transferred to the outer section at 12 h [25,26]. It was evidenced that hollow structures can also be obtained with the EG/Et ratio of 1:8 (Figure S2).

**Scheme 1:**
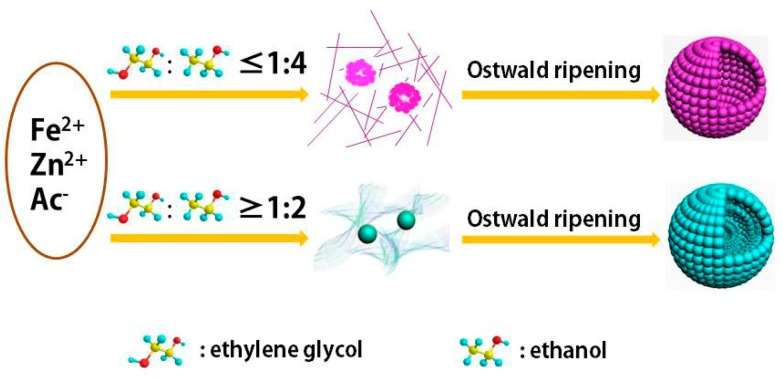
Schematic illustration for the formation mechanisms of hollow and solid ZnFe_2_O_4_ CNAs.

For the solid sphere system, the formation process was slightly different from that of the hollow sphere system. As depicted in Figure 4E,F, amorphous structures were formed when the synthetic time was no more than 1 h, and the particle sizes were very small (Figure S1B). When the synthesis time was extended to 2 h, loose aggregates with a large size distribution were observed. However, the XRD results (a and b in Figure S1B) showed that these samples were poorly crystalline. Pure zinc ferrite phase was obtained when the reaction was further prolonged to 4 h, and spherical structures with size of about 100 nm were obtained (Figure 4H). All of the peaks shown in XRD patterns (Figure S1B) can be matched to the standard pattern of the ZnFe_2_O_4_ phase (JCPDS No. 77-0011), which meant that pure ZnFe_2_O_4_ was formed when the synthetic time was 2 h in the solvothermal environment. The evolution of intermediates is schematically illustrated in Scheme 1.

### 2.4. Magnetic Characterization

**Figure 5:**
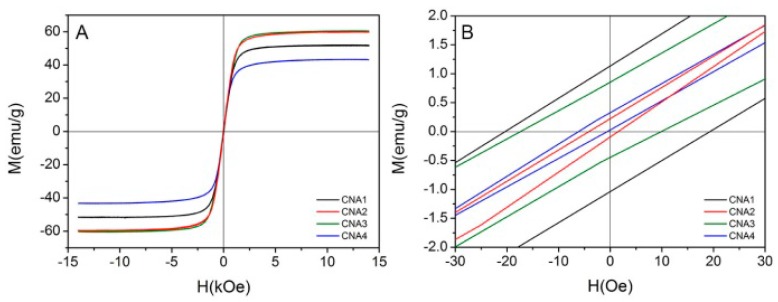
(**A**) Magnetization hysteresis curve of ZnFe_2_O_4_ CNAs; and (**B**) the magnified graph of (**A**).

The magnetization hysteresis curves for ZnFe_2_O_4_ CNAs were measured using a vibrating sample magnetometer at room temperature. Figure 5A showed the magnetization property of the samples, but it was hard to see the hysteresis loops at the full scale. As depicted in Figure 5B (the close-up view of Figure 5A), all of the samples presented a very small hysteresis loop, evidencing the weak ferromagnetic behavior of all the CNAs. The values of saturation magnetization (Ms) were measured to be 60.4, 60.3, and 43.2 emu·g^−1^ for CNA3, CNA2, and CNA4, respectively. It can be noted that the Ms increased accordingly with the crystallite size, in accordance with the results reported previously [4,27]. Clearly, the Ms values of CNA2 and CNA4 were rather different, which should be ascribed to the nature of the self-assembled structure of these two samples. CNA2 and CNA4 showed very low remnant and coercivity among all the samples, which indicated that the superparamagnetic–ferromagnetic transformation might occur with crystallite sizes slightly larger than 15 nm. These results could be partially attributed to size effects, such as surface disorder in small CNAs particles [28,29]. However, sample CNA1—hollow spheres with a crystallite size of 20.4 nm—exhibited a saturation magnetization of 52.0 emu·g^−1^, which was smaller than CNA2 with a crystallite size of 15.8 nm. This should be attributed to the unique hollow structure of CNA1 as well as its crystalline nature, size effect, and the arrangement of nanoparticles in the assemblies. Hollow spheres also exhibited a higher remnant of 1.31 emu·g^−1^ and coercivity of 22.0 Oe compared to that of 0.18 emu·g^−1^ and −0.2 Oe for CNA2, 0.83 emu·g^−1^ and 9.9 Oe for CNA3, and 0.35 emu·g^−1^ and 2.1 Oe for CNA4, respectively. Increasing the magnetic anisotropy is a typical method to improve the coercivity of a system. This anisotropy included magnetocrystalline, shape, or strain anisotropy [30]. It can be supposed that the ZnFe_2_O_4_ crystal in the hollow structure enlarged the anisotropy of the particles, which led to a high remnant and coercivity.

### 2.5. Adsorption of Congo Red

**Figure 6:**
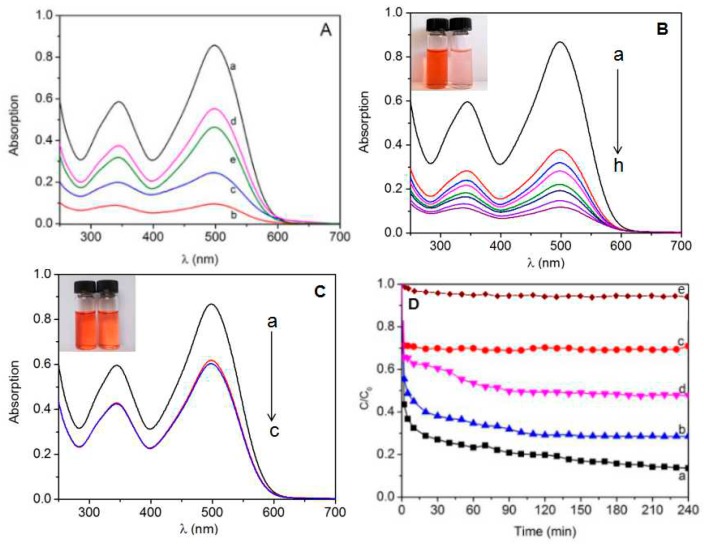
(**A**) The UV-Vis absorption spectra of aqueous solutions of Congo red (15 mg·L^−1^) at (a) 0 min and in the presence of ZnFe_2_O_4_ (b) CNA1, (c) CNA2, (d) CNA3, (e) CNA4 for 60 min; the variations of the absorption spectra of aqueous Congo red solutions in the presence of (**B**) hollow sphere (CNA1) at different time intervals of (a) 0, (b) 2, (c) 5, (d) 10, (e) 40, (f) 80, (g) 150 and (h) 240 min and (**C**) solid sphere (CNA3) at different time intervals of (a) 0, (b) 5 and (c) 240 min; and (**D**) the variations of adsorption rates of Congo red on (a) CNA1, (b) CNA2, (c) CNA3, (d) CNA4, and (e) in the absence of ZnFe_2_O_4_ CNAs under dark conditions. c_0_ (mg·L^−1^) is the initial concentration of the Congo red solution, and c (mg·L^−1^) is the Congo red concentration obtained after different time intervals during the adsorption.

Iron oxides have relatively high surface area and surface charge, which can be used to remove organic compounds in water through adsorption [31]. The iron oxide adsorbent can be separated from the medium by a simple magnetic process after the adsorption procedure [32,33,34]. The absorption activity of ZnFe_2_O_4_ CNAs was studied under dark conditions in aqueous organic dyestuff solutions of Congo red. Twenty milligrams of each CNA sample was taken to remove Congo red in 70 mL solutions with a concentration of 15 mg/L. As depicted in the UV-Vis absorption spectra in Figure 6A, the strength of the absorption peaks decreased with prolonged processing time, but the absorption peak positions did not change. Clearly, CNA1 showed the best absorption ability among all the samples, while CNA3 showed the worst. Namely, hollow ZnFe_2_O_4_ spheres can be used as a more efficient material for the treatment of waste organic water than the hierarchical solid spheres, which might benefit from their hollow structure to provide more accessible surface area. 

Figure 6D shows the variation of absorption rate of the CAN samples with processing time. Very fast removals of Congo red are observed within the first 2 min with the removals of about 56.5%, 44.6%, 34.3%, and 28.9% for CNA1, CNA2, CNA3, and CNA4, respectively. After the rapid decrease of the peak intensity for Congo red, the adsorption process became slow for all of the samples, with a removal of 29.9%, 26.9%, 1.7%, and 17.9% for CNA1, CNA2, CNA3, and CNA4 from 2 min to 4 h, respectively. The images and UV-Vis absorption spectra at different times of the best absorbent (CNA1) and the worst (CNA3) are respectively shown in Figure 6B,C. However, the absorption degree of CNA3 remained almost the same after 30 min, while the peak strength of CNA1 showed a steady decline until 210 min. From the curve of the change in c/c_0_ of Congo red in the absence of ZnFe_2_O_4_, a slight decrease during the test time can be observed due to the attachment on the container wall, but it only declines slightly when compared to the rapid and heavy decrease of the dye concentration caused by the significant absorption of ZnFe_2_O_4_ CNAs.

## 3. Discussion

**Figure 7:**
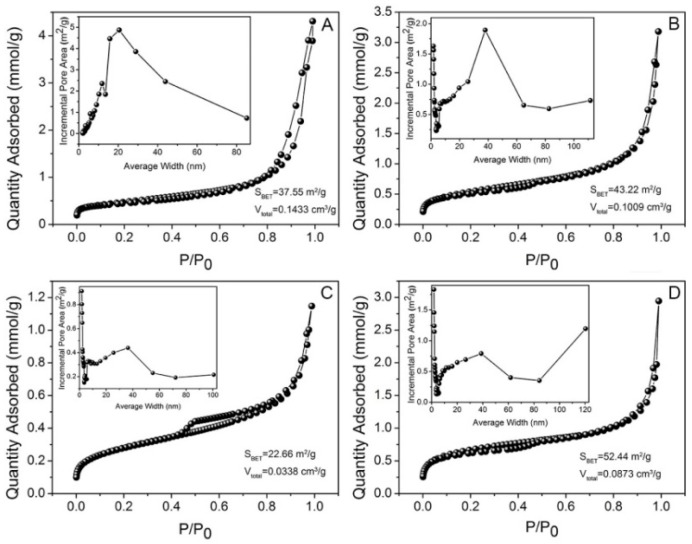
Nitrogen adsorption/desorption isotherms of (**A**) CNA1; (**B**) CNA2; (**C**) CNA3; and (**D**) CNA4. Insets show the Barrett–Joynet–Halenda (BJH) pore size distributions of CNAs.

The nature of the solvents and additives in synthetic systems for ferrite CNAs may play important roles in the determination of the structure of the final nanomaterials [4,21,35]. A polar solvent with a high boiling point should contribute to the formation of assembly structures via the in situ self-assembly of primary nanoparticles in the solvothermal synthesis, rather than the single-crystalline ferrite nanoparticles. The physicochemical properties—for example, magnetic and catalytic properties of ferrite CNAs—could be determined by both the sizes of primary ferrite nanoparticles and the structure of the final nanomaterials [21,27]. However, the specific surface area and the pore size are believed to be related to the adsorption performance [36,37]. As the size of the CNAs is submicrometer, the relatively higher density should also be conducive to its better adsorption ability. As depicted in Figure 7, the Brunauer–Emmett–Teller (BET) specific surface areas can be calculated to be about 37.6, 43.2, 22.7, and 52.4 m^2^·g^−1^ for CNA1, CNA2, CNA3, and CNA4, respectively. However, CNA1 and CNA2 had more proper pore volumes of 0.14 and 0.10 cm^3^·g^−1^, respectively, which made it easier for Congo red to penetrate into the spheres. This can be used to explain the high adsorption ability for Congo red and the different adsorption process after 2 min of all the CNA samples. The small surface area and pore volume of CNA3 were supposed to be the reason for the poor adsorption performance compared to that of CNA1 (Figure 7). Combined with their assembly structures, it should be helpful to explain why the adsorption performance of CNA3 reached equilibrium in 30 min while CNA1 took more than 3 h to do that.

## 4. Materials and Methods

The chemicals, including ZnCl_2_, FeCl_3_·6H_2_O, CH_3_COONa, Congo red, ethylene glycol (EG), and ethanol (Et) were of analytical grade (Sinopharm Chemical Reagent Company, Shanghai, China) and used as received. Double distilled water was used in the experiments.

In a typical synthesis, FeCl_3_·6H_2_O (2 mmol) and ZnCl_2_ (1 mmol) were dissolved in 30 mL mixed solvents with different EG/Et volume ratios and then mixed until homogeneous under vigorous stirring. Meanwhile, CH_3_COONa (5 mmol) was added to the mixture under vigorous stirring. The homogeneous mixture was transferred into a 40 mL Teflon-lined autoclave, which was then tightly sealed and heated at 200 °C for 12 h in an oven. The autoclave was then allowed to cool to room temperature naturally. Finally, the products were collected by centrifugation, washed separately with distilled water and ethanol several times, and dried in an oven at 60 °C for 6 h. 

Powder X-ray diffraction (XRD) patterns were conducted on a Bruker D8 Advance X-ray diffractometer (Bruker, Billerica, MA, USA) equipped with graphite monochromatized Cu Kα radiation (λ = 0.15418 nm) from 10° to 80° (2θ). The scanning electron microscopy (SEM) images were measured on a JSM-6390LV SEM with an operating voltage of 20 kV. Transmission electron microscopy (TEM) images were examined on a JEM2000EX TEM (JEOL Ltd., Tokyo, Japan) operating at 120 kV. Magnetic properties were measured using a LDJ9500 vibrating sample magnetometer (VSM) (LDJElectronics, Oakland, CA, USA) at room temperature. The Brunauer–Emmett–Teller (BET) surface area and pore diameter of the samples were determined from the N_2_ adsorption at −77 K using a TriStar 3000 system (Micromeritics, Norcross, GA, USA). Inductively coupled plasma-optical emission spectrometry (ICP-OES) was performed using an Optima8000 (PerkinElmer, Waltham, MA, USA) instrument.

The adsorption experiments of Congo red dye over the CNAs were performed in a quartz reactor under ultrasonication in dark conditions. The concentration of the dye was 15 mg·L^−1^, and a certain amount of CNAs was mixed into the solution. Then, the concentration of Congo red during the absorption was monitored by colorimetry using a TU1901 UV-Vis spectrometer (Purkingji General Instrument Limited Corporation, Beijing, China).

## 5. Conclusions

Hierarchical ZnFe_2_O_4_ CNAs were prepared solvothermally by controlling the constitution of the solvents. Hollow ZnFe_2_O_4_ spheres can be synthesized from synthetic systems with low EG/Et volume ratios, while solid ZnFe_2_O_4_ CNAs were obtained with the continuous increase of the EG content in the solvents. Experimental results showed that all the submicrometer CNAs with unique magnetic properties were formed by the ordered self-assembly of primary nanoparticles. The ZnFe_2_O_4_ CNAs could remove azo dye quickly and heavily in 2 min and be separated easily. These experimental results gave clues to the rational design of novel magnetic ferrite nanostructures with unique physicochemical properties.

## Supplementary Materials

The following are available online at www.mdpi.com/1996-1944/9/10/806/s1. Figure S1: XRD patterns of the intermediates prepared from the hollow (**A**) and solid ZnFe_2_O_4_ CNAs (**B**) systems: (a) 0.5 h, (b) 1 h, (c) 2 h and (d) 4 h. Figure S2: TEM image of ZnFe_2_O_4_ CNAs from the synthesis system with the volume EG/Et of 1:8.
