# Supplementary material for: Solvothermal Synthesis of Hierarchical Colloidal Nanocrystal Assemblies of ZnFe2O4 and Their Application in Water Treatment
Source: Materials (Basel). 2016 Sep 29;9(10):806. doi: 10.3390/ma9100806 (PMC5456613; doi:10.3390/ma9100806)
Supplement: Supplementary file 1 [file materials-09-00806-s001.pdf]

Supplementary Materials: Solvothermal Synthesis of Hierarchical Colloidal Nanocrystal Assemblies of $ZnFe_2O_4$ and Their Application in Water Treatment

Peizhi Guo, Meng Lv, Guangting Han, Changna Wen, Qianbin Wang, Hongliang Li and Xiusong Zhao

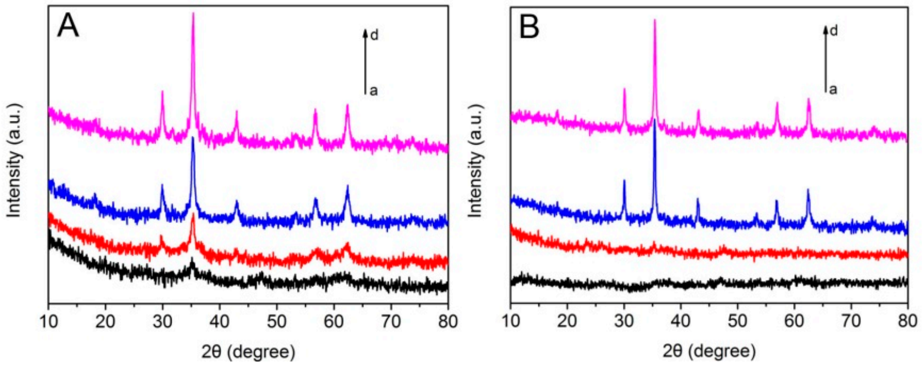

Figure S1. XRD patterns of the intermediates prepared from (**A**) the hollow $ZnFe_2O_4$; and (**B**) solid $ZnFe_2O_4$ systems: (a) 0.5 h, (b) 1 h, (c) 2 h, and (d) 4 h.

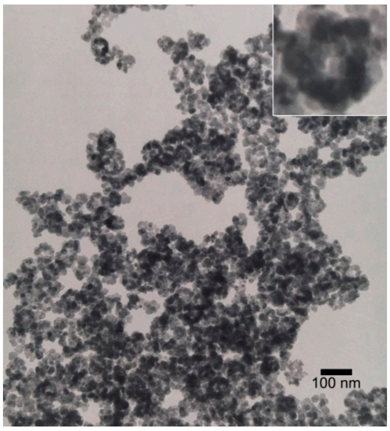

Figure S2. TEM image of $ZnFe_2O_4$ CNAs when the volume ethylene glycol/ethanol ratio (EG/Et) in the solvent is 1:8.
